# Risk estimation for the primary prevention of cardiovascular disease: considerations for appropriate risk prediction model selection

**DOI:** 10.1016/S2214-109X(24)00210-9

**Published:** 2024-07-17

**Authors:** Kim Robin van Daalen, Dudan Zhang, Stephen Kaptoge, Ellie Paige, Emanuele Di Angelantonio, Lisa Pennells

**Affiliations:** a British Heart Foundation Cardiovascular Epidemiology Unit, Department of Public Health and Primary Care, University of Cambridge, Cambridge, UK; b Victor Phillip Dahdaleh Heart and Lung Research Institute, University of Cambridge, Cambridge, UK; c British Heart Foundation Centre of Research Excellence, University of Cambridge, Cambridge, UK; d National Institute for Health and Care Research Blood and Transplant Research Unit in Donor Health and Behaviour, University of Cambridge, Cambridge, UK; e Health Data Research UK Cambridge, Wellcome Genome Campus and University of Cambridge, Cambridge, UK; f Population Health Program, QIMR Berghofer Medical Research Institute, Brisbane, QLD, Australia; g School of Public Health, University of Queensland, Brisbane, QLD, Australia; h Epidemiology and Population Health, The Australian National University, Canberra, ACT, Australia; i Health Data Science Research Centre, Human Technopole, Milan, Italy

## Abstract

Cardiovascular diseases remain the number one cause of death globally. Cardiovascular disease risk scores are an integral tool in primary prevention, being used to identify individuals at the highest risk and guide the assignment of preventive interventions. Available risk scores differ substantially in terms of the population sample data sources used for their derivation and, consequently, in the absolute risks they assign to individuals. Differences in cardiovascular disease epidemiology between the populations contributing to the development of risk scores, and the target populations in which they are applied, can result in overestimation or underestimation of cardiovascular disease risks for individuals, and poorly informed clinical decisions. Given the wide plethora of cardiovascular disease risk scores available, identification of an appropriate risk score for a target population can be challenging. This Review provides an up-to-date overview of guideline-recommended cardiovascular disease risk scores from global, regional, and national contexts, evaluates their comparative characteristics and qualities, and provides guidance on selection of an appropriate risk score.

## Introduction

Cardiovascular diseases are the number one cause of death globally, responsible for an estimated 19·8 million deaths in 2022,^1^ of which most occurred in low-income and middle-income countries (LMICs).^1^, ^2^ The underlying disease processes contributing to atherosclerotic cardiovascular disease typically commence years before acute cardiovascular disease events and clinical diagnosis.^3^ Identifying individuals at high risk and intervening to reduce risk before a cardiovascular disease event occurs underpins the majority of national and international primary prevention guidelines. Clinical guidelines for cardiovascular disease prevention commonly recommend using risk scores or prediction models, which assess the combined influence of multiple measured risk factors, to estimate future disease risk and identify people most likely to benefit from preventive interventions. Risk scores based on a combination of risk factors are more accurate at predicting cardiovascular disease risk than those that assess risk based on individual risk factors alone.^4^, ^5^ A plethora of cardiovascular disease risk scores have been developed and updated over the past five decades (eg, Framingham,^6^ Pooled Cohort Equations [PCE],^7^ Systematic Coronary Risk Evaluation [SCORE],^8^ SCORE2,^9^ WHO cardiovascular disease risk,^10^ PREDICT,^11^ QRISK3,^12^ and Predicting Risk of Cardiovascular Disease Events [PREVENT]^13^, ^14^).^15^ Different cardiovascular disease guidelines (eg, guidelines of the American College of Cardiology–American Heart Association,^5^ European Society of Cardiology,^4^ National Institute for Health and Care Excellence,^16^ and WHO^17^) recommend the use of distinct risk scores based on apparent suitability, as well as feasibility and practicability in local practice.^18^ With different risk scores being proposed for use, many of which appear to use the same risk factors, it can be challenging for health professionals and policy makers to understand the differences between them, and select the most appropriate risk score for their target population.

When using a risk score in practice, the estimated risk depends not only on the individual's measured risk factor values, but also on two additional risk model-specific features: the population average risk (incidence or mortality) and the relative risks (or associations) that confer the change in risk with differing levels of each measured risk factor. For example, a relative risk of 1·2 for a lipid measurement in a prediction model would confer a 1·2-fold increase in estimated risk per unit increase in the individual's measured value of that risk factor.

As the relative effect of risk factors on cardiovascular disease has been shown to be similar across different populations (ie, the association is similar across studies, countries, and time periods), different cardiovascular disease risk scores often result in a similar ranking of individuals based on their estimated risk.^10^, ^19^ However, population average risks might differ for several reasons, yet are often built into risk scores at development, leading to variation in risk estimates obtained across different risk scores, for a given set of measured risk factor values.^19^ Such variation might reflect the number of endpoints included in the cardiovascular disease definition, as well as the overall underlying risk of the population (data source) used to derive the risk prediction model. Many scores (in their original form) might not be appropriately calibrated to predict the correct level of absolute risk in the broader target population in which they are applied, resulting in overprediction or underprediction of cardiovascular disease risk. This miscalibration can affect the proportion of people assigned preventive intervention using a given risk threshold and the perceived severity of risk by the individual and clinician using the risk score. Such miscalibrations might occur due to geographical and temporal differences in cardiovascular disease event rates and risk factor levels between study populations used to derive the model and the target populations. Miscalibration can be fixed by recalibrating the model to the target population; however, many risk scores are assigned for use in target populations without consideration of this step. This issue is particularly relevant to LMICs where cardiovascular disease rates are not only currently estimated to be high and changing rapidly,^20^ but where there is also a paucity of nationally representative cohorts with often no validated population-specific cardiovascular disease risk assessment tools developed using prospective data.^21^, ^22^ A solution is using a risk score derived using data from countries where studies and data are plentiful, but conducting careful recalibration with available routine data sources to ensure reasonably accurate risk predictions for geographically distinct target populations.

Previous reviews of cardiovascular disease risk scores have outlined criteria for clinically useful risk estimation models and many have summarised the numerous scores that have been developed, with some providing quantitative evaluations and others discussing limitations and advantages.^15^, ^23^, ^24^, ^25^ Nevertheless, an update is warranted as cardiovascular epidemiology is rapidly evolving and novel risk model development continues. Furthermore, few reviews have explicitly considered whether potential risk scores have specifically and systematically ensured adequate calibration of the score for the contemporary target population, as well as making provision for recalibration approaches to allow continual updates in the future. Therefore, this Review presents a guiding framework on factors to be taken into consideration by health professionals and policy makers when selecting a cardiovascular disease risk model for implementation in clinical practice, including crucially the ability to recalibrate, and provides an up-to-date overview of the main guideline-recommended cardiovascular disease risk scores from global, regional, and national contexts, and summarises their qualities.

## Criteria for cardiovascular disease risk prediction model selection

Many criteria should be considered when selecting an appropriate cardiovascular disease risk score for clinical use. Key criteria are outlined in table 1, which includes consideration of practical, technical, and methodological aspects of the risk score development and validation.^26^, ^27^ Existing guidelines for risk score derivation and validation (eg, TRIPOD^28^ and PROBAST^29^) provide in-depth description and discussion of statistical criteria and should be consulted in addition to the current overview. Likewise, in-depth guidance, which goes beyond the scope of this Review, on metrics that can be used to assess predictive performance is given elsewhere.^27^ A summary of the main guideline-recommended risk scores from global, regional, and national contexts are provided in table 2 and table 3.

**Table 2:** Comparison of global, regional, and subregional cardiovascular disease risk prediction scores

		**WHO cardiovascular disease risk**^10^	**WHO–International Society of Hypertension**^30^	**Globorisk**^31^	**INTERHEART modifiable risk score**^32^	**SCORE**^8^	**SCORE2**^9^	**SCORE2-OP**^33^
Derivation								
	Population	85 prospective cohorts with 376 177 individuals and 19 333 cardiovascular disease events; participant age range 40–80 years; baseline survey conducted in period 1960–2013; located in Europe, North America, Japan, and Australia	No single derivation cohort; risk factor distribution, relative risks, and cardiovascular disease incidence from various sources combined; incidence-based location, risk factors from 14 WHO regions	Eight prospective cohorts with 50 129 individuals and 6042 cardiovascular disease events; participant age range 40–84 years; baseline survey conducted in period 1948–1993; located in North America	Case control study with 5349 participants and 7423 control participants; participant median age 58 years (range 49–67); baseline survey conducted in period 1999–2003; located in 52 countries	12 prospective cohorts with 205 178 individuals and 7934 fatal cardiovascular disease events; participant age range 40–65 years; baseline survey conducted in period 1967–1991; located in Europe	45 prospective cohorts with 677 684 individuals and 30 121 cardiovascular disease events; participant age range 40–69 years; baseline survey conducted in period 1990–2009; located in Europe	Prospective cohort; participants older than 65 years; baseline survey conducted in period 1994–2003; located in Europe
	Risk factors (laboratory version)	Age, sex, smoking, systolic blood pressure, total cholesterol, diabetes	Age, sex, smoking, systolic blood pressure, total cholesterol, diabetes	Age, sex, smoking, systolic blood pressure, total cholesterol, diabetes	Age, sex, smoking, diabetes, hypertension, ratio of apolipoprotein B to apolipoprotein A1, or ratio of total cholesterol to HDL cholesterol	Age, sex, smoking, systolic blood pressure, total cholesterol or ratio of total cholesterol to high-density lipoprotein cholesterol	Age, sex, smoking, diabetes, systolic blood pressure, ratio of total cholesterol to high-density lipoprotein cholesterol	Age, smoking, diabetes, systolic blood pressure, ratio of total cholesterol to high-density lipoprotein cholesterol
	Risk factors (non-laboratory version)	Age, sex, systolic blood pressure, smoking, BMI	Sex, age, systolic blood pressure, smoking, diabetes	Age, sex, smoking status, systolic blood pressure, BMI	Age, sex, smoking, diabetes, hypertension, family history of myocardial infarction, diet, lifestyle, psychosocial factors	NA	NA	NA
	Outcomes and timeframe, when applicable	10-year risk of fatal and non-fatal cardiovascular disease (ie, coronary heart disease or stroke)	10-year risk of fatal and non-fatal cardiovascular disease (ie, coronary heart disease or stroke)	10-year risk of fatal and non-fatal cardiovascular disease (ie, coronary heart disease or stroke)	Risk of fatal and non-fatal myocardial infarction	10-year risk of fatal cardiovascular disease (ie, coronary heart disease or stroke)	10-year fatal and non-fatal cardiovascular disease outcomes	5-year and 10-year fatal and non-fatal cardiovascular disease outcomes
	Follow-up	Over 10-year follow-up in most cohorts	No actual follow-up, hypothetical 10-year	Over 10-year follow-up in seven of eight cohorts	No follow-up, case–control design	Over 10-year follow-up in all cohorts	Median follow-up 10·7 years (5th–95th percentile 5·0–18·6)	Median 13 years (IQR 8–15)
	Statistical model	Cox survival models	Combination of several relative and absolute risks in Cox model-type structure	Cox survival model	Unconditional logistic regression	Weibull survival models	Fine and Gray competing risk-adjusted models	Fine and Gray competing risk-adjusted models
Internal validation		Well validated internally	Not applicable	Well validated internally	Well validated internally	Well validated internally	Well validated internally	Well validated internally
External validation		Well validated in several external cohorts	No adequate external validation	Well validated in several external cohorts	Well validated in several external cohorts	Well validated in several external cohorts	Well validated using data from 25 prospective cohorts	Validated in six studies
Implementation								
	Format	Colour-coded charts and software code; online calculator to follow	Colour-coded charts	Colour-coded charts; online calculator	Calculation charts	Colour-coded charts and online calculator	Colour-coded charts and online calculator	Colour-coded charts and online calculator
	Low-resource setting	Yes	Yes	Yes	Yes	No	No	No
	Country-specific versions available?	Different charts for 21 worldwide regions	Different charts for 14 worldwide regions	Different charts available per country	NA	Low-risk charts and high-risk charts, for grouped European countries	Low-risk, moderate-risk, high-risk, and very high-risk charts, for grouped European countries	Low-risk, moderate-risk, high-risk, and very high-risk charts, for grouped European countries
	Guidelines	WHO guidelines on cardiovascular disease prevention 2019	WHO guidelines for cardiovascular disease prevention 2007	NA	NA	2019 ESC Guidelines	2021 ESC Guidelines on cardiovascular disease prevention in clinical practice	2021 ESC Guidelines on cardiovascular disease prevention in clinical practice
Recalibration								
	Recalibrated?	Integral part of model development; calibrated to 21 global regions	Integral part of construction; calibrated for 14 global regions	Integral part model development; calibrated for 187 countries	Not recalibrated for different settings	High-risk and low-risk charts provided; recalibrated post hoc in several countries	Integral part of model development; calibrated to risk regions	Integral part of model development; calibrated to risk regions
	Recalibration data	Region-specific incidences from GBD and country-specific risk factors from NCD-RisC	Risk factor distributions from WHO Comparative Risk Assessment study; region-specific incidence from GBD	Used country-specific mortality rates and case-fatality to estimate incidence rates based on age trends in Swedish data; rates then modelled over past years and projected into future 10 years	NA	Country-specific cohorts	Country-specific risk factors from NCD-RisC; cardiovascular disease mortality rates from WHO; multipliers used to convert cardiovascular disease mortality to total cardiovascular disease incidence based on representative data from each region	Country-specific risk factors from NCD-RisC; cardiovascular disease mortality rates from WHO; multipliers used to convert cardiovascular disease mortality to total cardiovascular disease incidence based on representative data from each region
	Recalibration methods	Simple framework and statistical code provided; can be applied using routinely available data	No specific method provided	Intuitive framework provided; can be applied with routinely available data; requires modelling if future projections of rates desired as used in original score	NA since model does not predict absolute risk	No standardised approach recommended	Framework and statistical code provided; can be applied using routinely available data	Framework and statistical code provided; can be applied using routinely available data
Advantages		Risk prediction charts provided for different ethnic-geographical regions; simplicity of recalibration approach with code provided to allow efficient updating; a non-laboratory variant is available; risk distribution illustrated for 79 countries	Risk prediction charts provided for different ethnic-geographical regions; non-laboratory variant is available	Risk prediction charts provided for different ethnic-geographical regions; intuitive systematic recalibration approach; a non-laboratory variant is available	Included large number of women, youth, and people from low-income and middle-income countries in derivation; a non-laboratory variant is available	Existence of low-risk and high-risk charts for European countries	Based on large dataset with European data; function has been recalibrated to four risk regions of Europe using representative mortality and incidence data; can be readily recalibrated to country and with updated data in the future; adjusted or competing risk of non-cardiovascular disease death; risk distribution illustrated for all ESC states	Function has been recalibrated to four risk regions of Europe using representative mortality and incidence data; can be readily recalibrated to country and with updated data in the future; specific function for older adults; calculates both 5-year and 10-year risk; adjusted or competing risk of non-cardiovascular disease death
Limitations		Data used in model derivation were mostly from high-income countries	Absence of individual-level population data; models based on summary inputs; no internal or external validation of the model in epidemiological cohorts	Rates used in recalibration rely on many modelling steps, assumptions, and projections; only USA-based data in model derivation	Case–control rather than cohort design used; might induce bias in relative risk estimates, prevents estimation of absolute risk; not available for different settings	Only estimates risk of fatal cardiovascular disease; no non-laboratory variant available; model derived only in European cohorts	No non-laboratory variant available; model derived only in European cohorts	No non-laboratory variant available; model derived only in European cohorts

ESC=European Society of Cardiology. GBD=Global Burden of Disease study. NA=not applicable. NCD-RisC=Non-Communicable Disease Risk Factor Collaboration.

**Table 3:** Comparison of national cardiovascular disease risk prediction scores

		**PREDICT**^11^	**ASSIGN**^34^	**QRISK 3**^12^	**PROCAM**^35^, ^36^	**China-PAR**^37^	**PCE**^7^	**Framingham**^6^	**Reynolds (men)**^38^	**Reynolds (women)**^39^	**PREVENT**^13^, ^14^
Derivation											
	Population	Prospective cohort study of PREDICT study with 401 752 individuals and 15 386 cardiovascular disease events; participant age range 30–74 years; baseline survey conducted in period from August, 2002, to October, 2015; located in New Zealand	Scottish Heart Health Extended Cohort prospective cohort study with 13 297 individuals and 2619 cardiovascular disease events; participant age range 30–74 years; baseline survey conducted in period 1984–87; located in Scotland	Prospective cohort study of QResearch database version 41 with 7·89 million general practice attendees and 363 565 events; participant age range 25–84 years; baseline survey conducted in period 1998–2015; located in the UK	Prospective cohort of healthy employee volunteers with 26 975 individuals, 511 events (coronary), and 8130 individuals, 85 events (stroke); participant age range 20–75 years; baseline survey conducted in period 1978–1995; located in Germany	Two prospective cohorts (InterASIA and China Multi-Center Collaborative Study of Cardiovascular Epidemiology 1998) with 21 320 individuals and 1048 cardiovascular events; participant age range 35–74 years; baseline survey conducted in 1998; located in China	Four prospective cohort studies (ARIC, CHS, CARDIA, Framingham Original and Offspring cohort) with 24 626 individuals and 2689 cardiovascular events; participant age range 20–79 years; baseline survey conducted in period 1984–93; located in the USA	Prospective cohort studies (Framingham Heart and Offspring) with 8491 individuals and 1174 cardiovascular disease events; participant age range 30–75 years; baseline survey conducted in periods 1968–1971, 1971–75, and 1984–87; located in the USA	Prospective cohort of Physicians Health Study II with 10 724 individuals and 1294 cardiovascular disease events; participant age range ≥50 years; baseline survey conducted in December, 1995; located in the USA	Prospective cohort of Women's Health Study with 24 558 individuals, 766 cardiovascular disease events; participant age range ≥45 years; baseline survey conducted in September, 1992; located in the USA	25 prospective cohort and electronic medical record datasets with 3 281 919 individuals, 106 661 total cardiovascular disease events (66 503 atherosclerotic cardiovascular disease, 59 350 heart failure, 35 980 coronary heart disease, 33 160 stroke); participant age range 30–79 years; baseline survey conducted in period 1992–2017; located in the USA
	Risk factors (laboratory version)	Age, sex, self-identified ethnicity, smoking, family history of premature cardiovascular disease, diabetes, systolic blood pressure, ratio of total cholesterol to HDL cholesterol, New Zealand Index of Socioeconomic Deprivation, atrial fibrillation, blood pressure-lowering, lipid-lowering, or antithrombotic medication	Age, sex, total cholesterol, HDL cholesterol, systolic blood pressure, smoking, number of cigarettes smoked per day, diabetes, social deprivation, family history of coronary heart disease	Age, ethnicity, deprivation (measured by the Townsend score, where higher values indicate higher levels of material deprivation), systolic blood pressure, BMI, total cholesterol, HDL cholesterol, smoking, family history of coronary heart diseases, type 1 diabetes, type 2 diabetes, treated hypertension, rheumatoid arthritis, arterial fibrillation, chronic kidney disease, systolic blood pressure variability, migraine, corticosteroids, systemic lupus erythematosus, atypical antipsychotics, severe mental illness, HIV or AIDs	LDL cholesterol, HDL cholesterol, systolic blood pressure, smoking, triglycerides, diabetes, family history of cardiovascular disease (coronary); age, sex, diabetes, smoking systolic blood pressure (stroke)	Age, systolic blood pressure, smoking, diabetes, total cholesterol, HDL cholesterol, treatment for systolic blood pressure, waist circumference, urbanisation (urban or rural), geographical region, family history of cardiovascular disease only for men	Age, sex, race, total cholesterol, HDL cholesterol, systolic blood pressure, antihypertensive treatment, diabetes, smoking	Age, sex, total cholesterol, HDL cholesterol, systolic blood pressure, smoking, diabetes, hypertensive treatment	Age, systolic blood pressure, high-sensitivity C-reactive protein, total cholesterol, HDL cholesterol, smoking, family history of myocardial infarction before 60 years old	Age, systolic blood pressure, high-sensitivity C-reactive protein, total cholesterol, HDL cholesterol, haemoglobin A1c if diabetic, smoking, family history of myocardial infarction before 60 years old	Age, sex, systolic blood pressure, smoking, total cholesterol, anti-hypertensive or statin use, diabetes, estimated glomerular filtration rate; optional for cardiovascular disease sub-type: urine albumin-creatinine ratio, haemoglobin A1C, social deprivation index
	Risk factors (non-laboratory version)	Age, sex, BMI, systolic blood pressure, smoking, diabetes, hypertensive treatment	NA	NA	NA	NA	NA	Age, sex, BMI, systolic blood pressure, smoking, diabetes, hypertensive treatment	NA	NA	NA
	Outcomes and timeframe	5-year risk of fatal and non-fatal cardiovascular disease	10-year risk of fatal and non-fatal cardiovascular disease	10-year risk of fatal and non-fatal cardiovascular disease events	10-year risk of major coronary events and cerebral ischaemic events	10-year risk of first atherosclerotic cardiovascular disease event	10-year risk of first atherosclerotic cardiovascular disease event (coronary heart disease death, non-fatal myocardial infarction, and fatal or non-fatal stroke)	10-year risk of fatal and non-fatal coronary heart disease, cardiovascular disease, myocardial infarction, and stroke	10-year risk of myocardial infarction, ischaemic stroke, coronary revascularisation, and cardiovascular death (coronary heart disease and cardiovascular disease combined)	10-year risk of myocardial infarction, ischaemic stroke, coronary revascularisation, and cardiovascular death (coronary heart disease and cardiovascular disease combined)	10-year and 30-year risk of fatal and non-fatal cardiovascular disease (atherosclerotic cardiovascular disease, heart failure)
	Follow-up	Mean 4·2 years, one third of participants over 5 years	Over 10-year follow-up	Median 4·4 years, around 1 million patients over 10-year period	Over 10-year follow-up	Average 12·3 years	Over 12-year follow-up	>10-year follow-up	Median 10·8 years (IQR 7·9-11·2)	Median 10·8 years (IQR 7·9-11·2)	Mean 4·7 years (SD 3·3)
	Statistical Model	Cox survival models	Cox survival models	Cox survival models	Cox survival models and Weibull survival models	Cox survival models	Cox survival models	Cox survival models and Weibull survival models	Cox survival models	Cox survival models	Cox survival models
Internal validation		Well validated internally	Well validated internally	Well validated internally	Well validated internally	Well validated internally	Well validated internally	Well validated internally	Well validated internally	Well validated internally	Well validated internally
External validation		Not externally validated yet	Not validated in non-Scottish population	QRISK 3 not externally validated yet; QRISK 1 and QRISK 2 externally validated in several cohorts	Well validated in several external cohorts	Validated in several external cohorts (China-PAR, Fangshan cohort study, Inner Mongolian)	Well validated in several external cohorts	Well validated in several external cohorts	Well validated in several external cohorts	Well validated in several external cohorts	Well validated with an additional 21 datasets
Implementation											
	Format	An electronic decision support system integrated within primary care patient management systems	Online calculator	Online calculator	Online calculator and simple scoring sheet	Online calculator and application (programme)	Online calculator, and downloadable spreadsheet	Online calculator, simplified scoring sheet, colour-coded chart, portable calculator	Online calculator	Online calculator	Online calculator
	Low-resource setting	No	No	No	No	No	No	Yes	No	No	No
	Guidelines	2018 Cardiovascular Disease Risk Assessment and Management for Primary Care (New Zealand)	SIGN	NICE guidelines on lipid modification, Joint British Societies Guidelines	International Task Force for Prevention of Coronary Disease guidelines	Guideline on the assessment and management of cardiovascular risk in China 2019	2019 ACC–AHA Guideline on the Primary Prevention of Cardiovascular Disease	NCEP guidelines, Canadian cardiovascular disease guidelines, other national guidelines recommending adapted versions	2010 ACCF–AHA guideline for assessment of cardiovascular risk in asymptomatic adults	Not part of design	Recommended in AHA published statement
Recalibration		Not part of design but data should be representative of target population	Not part of design	Not part of design, but data should be representative of target population	Not part of design	Not part of design	Not part of design	Not part of design, but has been completed for different countries	Not part of design	High-sensitivity C-reative protein and family history of myocardial infarction before 60 years old were included to improve the discrimination ability of equations	Not part of design
Advantages		Derived in a large contemporary cohort; minimal missing data derivation dataset; socioeconomic status, ethnicity, and several other variables are added to help identify high-risk patient groups who might otherwise be undertreated	Inclusion of deprivation index and family history of cardiovascular disease	Big population-derived dataset; scores based on general practice registers have the potential for ongoing revision; score estimates risk specifically for a UK population		Waist circumference, geographical region, urbanisation, and family history of atherosclerotic cardiovascular disease were included to improve the discrimination ability of equations	Derivation cohorts broadly representative of the population of the USA	A non-laboratory variant is available; multi-generational dataset with highly complete data	High-sensitivity C-reactive protein and family history of myocardial infarction before 60 years old were included to improve the discrimination ability of equations	No non-laboratory variant available; derivation datasets are not diverse in terms of ethnicity, socioeconomic status, and health status, which might limit generalisability and calibration	Big, contemporary, and diverse population derived dataset; inclusion of social deprivation index as a widely available measure of area-based deprivation
Limitations		No non-laboratory variant available; not well externally validated	No non-laboratory variant available; no risk prediction for different ethnic-geographical regions; not well validated	No non-laboratory variant available; substantial amount of missing data; imputation and statistical modelling to reduce bias; non-standardised measurement of outcomes	No non-laboratory variant available; derivation sample might not be representative for the general population; score might be underpowered for women	No non-laboratory variant available; cohorts represent risk in China from two decades ago; not generalisable to the various incidence rate, risk-factor levels, and composition of cardiovascular disease in different regions in China	No non-laboratory variant available; cohorts used in derivation are not current	Data used in model derivation potentially not nationally representative	No non-laboratory variant available; derivation datasets are not diverse in terms of ethnicity, socioeconomic status, and health status, which might limit generalisability and calibration	Online calculator	No non-laboratory variant available; individual-level social determinants not routinely available for all datasets

ACC-AHA=American College of Cardiology–American Heart Association. ARIC=Atherosclerosis Risk in Communities study. ASSIGN=Assessing Cardiovascular Risk to Scottish Intercollegiate Guidelines Network. CARDIA=Coronary Artery Risk Development in Young Adults. CHS=Cardiovascular Health Study. NCEP=National Cholesterol Education program. NICE=National Institute for Health and Care Excellence. PREDICT=Prospective Evaluation of Diabetic Ischaemic Disease by Computed Tomography. PREVENT=AHA Predicting Risk of cardiovascular disease Events. PROCAM=Prospective Cardiovascular Münster epidemiology study. SIGN=Scottish Intercollegiate Guidelines Network.

### Dataset used for model development

Ideally, cardiovascular disease risk scores are developed using data from large single or pooled prospective cohort studies (eg, SCORE,^8^ SCORE2,^9^ PCE,^7^ WHO cardiovascular disease risk,^10^ and PREVENT^13^, ^14^). This development approach enables reliable estimation of risk ratios based on measured risk factor values and estimation of absolute risk over a specific timeframe (eg, 10 years). Contrastingly, case–control data are less suitable, not only due to bias in approximating risk ratios with odds ratios, but also due to the unsuitability of case–control study designs for incidence estimation, preventing absolute risk estimation without further modelling steps and assumptions (eg, INTERHEART modifiable risk score).^32^ Health-care data accessible through electronic health records have become common data sources to establish open cohorts used for risk prediction. For example, the QResearch general practice database in the UK has enabled the derivation and continual updating of the QRISK models,^12^, ^40^, ^41^ based on a substantially large and rich dataset (eg, the QRISK3 update included 7·89 million attendees^12^). Although the use of such electronic health records data is promising in many cases, several factors should be kept in mind when considering the validity of risk models developed using such data: data collection is often not standardised, risk factors are obtained at different timepoints, and missing data are commonly substantial and preferentially among healthier or younger individuals who interact less with the health-care system, or population groups with reduced health-care access or help-seeking behaviours.^42^, ^43^, ^44^ The New Zealand PREDICT database has overcome some of these limitations, covering around 90% of the eligible population of New Zealand with few missing data.^11^ PREVENT uses a combination of both research cohorts and electronic health records datasets, demonstrating consistent risk associations between risk factors and cardiovascular disease in both data types. Using the large, contemporary, and diverse sample of electronic health records data covering all census regions adds to the real-world representativeness and generalisability of the cardiovascular disease risk estimates.^13^, ^14^

### Risk factors included in cardiovascular disease risk models

Most cardiovascular disease risk prediction scores include an established set of risk factors such as age, sex, smoking, systolic blood pressure, total cholesterol, and HDL cholesterol. Most scores also include type 2 diabetes (eg, PCE,^7^ WHO cardiovascular disease risk,^10^ Globorisk,^31^ PREVENT^13^, ^14^), whereas others assume separate risk scores will be used for those with diabetes (eg, SCORE,^8^ SCORE2^9^). Additional factors, such as medication use, family history of cardiovascular disease, social deprivation, or specific pre-existing conditions or disease markers (eg, Assessing Cardiovascular Risk using SIGN Guidelines [ASSIGN]*,*^34^ PREVENT,^13^, ^14^ PREDICT,^11^ QRISK3^12^), might also be included and can improve discrimination. Although the inclusion of additional predictors might appear to provide small incremental improvement in discrimination for the whole population, some factors might provide more accurate risk estimation for some subgroups (eg, estimated glomerular filtration rate is more predictive for individuals who have chronic kidney disease than those who do not).^45^, ^46^, ^47^ Traditional global measures of discrimination might be limited in showing such benefits and additional consideration of risk predictor hazard ratios, differences in resulting absolute risk predictions, and risk reclassification for affected individuals might be warranted.^47^ When choosing a cardiovascular disease risk score, whether the health-care professional has the facility to measure all the included risk factors should also be evaluated. Availability of risk factor measurements in the target population is also an important consideration for recalibration of the risk score. The development of non-laboratory versions of several risk scores (eg, Framingham, WHO cardiovascular disease risk*,*^10^ WHO–International Society of Hypertension,^30^ Globorisk,^31^ INTERHEART modifiable risk score^32^) enables their use in a wide range of communities (eg, settings that have low access to medical facilities).^22^

### Cardiovascular disease endpoint definitions

Cardiovascular disease risk scores can differ substantially in the definitions of predicted endpoints, including variations in the timeframe of risk prediction (eg, 5-year, 10-year, 30-year, lifetime risk), disease outcome (eg, total cardiovascular disease, stroke, myocardial infarction risk) and fatality (eg, total incidence, mortality). Most current guidelines recommend using 10-year risk assessments for general populations. Lifetime risk, the risk of developing cardiovascular disease during a person's remaining lifetime, has been suggested for use in younger individuals (ie, individuals younger than 50 years) and for older adults (ie, individuals older than 75 years).^48^ Younger individuals might have a low 10-year risk (due to their age), but a high lifetime risk due to a more extreme risk factor burden. Therefore, lifetime risk can be used to recommend lifestyle changes in younger individuals to reduce cardiovascular disease risk.^49^

Most current scores calculate total incidence (fatal and non-fatal) cardiovascular disease, whereas those disregarding non-fatal events (eg, morbidity) only predict fatal cardiovascular disease risk (eg, SCORE^8^). The latter has limitations as most first-time cardiovascular disease events are non-fatal (depending on the target populations and access to medical facilities). Risk scores predicting only mortality can underestimate the total cardiovascular disease burden, particularly in populations where case-fatality is low (eg, younger individuals) and can thereby skew treatment recommendations towards older adults.^9^ Thus, predicting fatal outcomes limits the effective targeting of preventive action to a small population subset.

Lastly, different cardiovascular disease risk scores might predict somewhat different collections of cardiovascular disease endpoints. Most include myocardial infarction and strokes, but they tend to differ in the inclusion of additional vascular-related endpoints, such as angina or revascularisations. When choosing a risk score, it is important to consider different endpoints, to choose those most applicable to the target population and to adjust intervention thresholds accordingly. More definitive endpoints requiring admission to hospital (eg, myocardial infarction, stroke) tend to be more consistently and accurately captured across populations, which can be beneficial if there is a need for score recalibration using population statistics. However, focusing on such endpoints could lead to underestimating the broader cardiovascular disease burden. This matter should be carefully considered when embedding a risk score into policy to ensure that the full preventive potential is realised.

### Statistical model used for model development

Most cardiovascular disease risk scores have been derived using survival analysis models, which are appropriate to allow for variable observation time and losses to participant follow-up within the cohort. Most risk scores use semiparametric Cox survival models (eg, ASSIGN,^34^ QRISK3,^12^ PCE,^7^ Reynolds,^38^, ^39^ Cardiovascular Disease Risk in China [China-PAR],^37^ WHO cardiovascular disease,^10^ WHO–International Society of Hypertension,^30^ Globorisk,^31^ PREVENT^13^, ^14^), although some use the parametric Weibull survival model (eg, SCORE^8^). The choice between Cox and Weibull makes little practical difference for hazard ratio estimations, but Weibull makes assumptions regarding to the shape of underlying hazard function whereas Cox does not. The new SCORE2,^9^ SCORE2-OP,^33^ and PREVENT^13^, ^14^ models allow for competing risks of non-cardiovascular disease deaths, thereby avoiding risk overestimation in populations with common competing events (eg, older adults).^9^ The INTERHEART modifiable risk score used data from a case-control study and unconditional logistic regression and is therefore unable to provide absolute risk estimates in the incidence framework.^32^

Machine learning-based or artificial intelligence (AI)-based prediction models have gained popularity over the past decade, with advocates stating that these agnostic approaches can provide greater modelling flexibility than traditional approaches.^44^ However, although AI-based prediction models show some advantages for dealing with high-dimensional data, large model development datasets are needed to reduce the instability of individual predictions and ensure the generalisability of the risk models (ie, to avoid overfitting). Research shows that, without the benefit of a considerable sample size, different samples from the same overarching population could result in very different models and individual predictions, even when the same model development methods are being used.^26^, ^50^ Furthermore, there might be barriers to the practical implementation of machine learning-based models given the extent of information to be elicited, and a so-called black box style application might reduce trust and uptake by potential users.^44^ To our knowledge, thus far, no AI-based cardiovascular disease clinical prediction model has been recommended by guidelines for primary cardiovascular disease prevention. TRIPOD and PROBAST provide details on appropriate analysis methods to ensure minimal bias and good applicability of risk models,^28^, ^29^ and specific TRIPOD-AI^51^ and PROBAST-AI guidelines are under development for the reporting of clinical prediction models that use regression or machine learning methods.^52^

### Performance of risk scores: internal and external validation

In general, to assess the performance of a prediction model, three key criteria should be considered in quantitative assessment: the association of predictors with outcomes; adequate discrimination (ie, ability of a model to predict who will have a cardiovascular disease event first; eg, using Harrell's C-index); and adequate calibration (ie, agreement between predicted and observed risk). Measures of overall fit and measures of clinical utility can be further used to reinforce findings of adequate predictive ability. Where sample sizes are smaller, overfitting should be checked using bootstrapping, cross-validation, and shrinkage approaches.^53^ Where it is of interest to compare different risk scores with alternative risk predictor inclusion, reclassification assessment might be relevant.^54^ Such comparison is of most relevance where there are predefined risk categories and individuals in the dataset are representative of the target population for screening pertaining to their risk distribution.

External validation can be applied to check the transferability of the risk score (model risk ratios) to a new, or broader population, completely distinct from the derivation data source.^22^, ^23^ This validation should include assessing how well the model predicts those who develop cardiovascular disease in the target population. Assessment of calibration is only relevant in an external dataset if the individuals are truly representative of the target population for screening. However, accurate representation might be seldom because cohort studies commonly represent a past time period and population subsets.

All scores compared in this Review (table 2; table 3) have been well internally validated, with C-statistics suggesting good or acceptable discrimination ability. The extent of external validation varies by risk score, how long it has been available, and the ease of calculation in external datasets. The Framingham risk score,^6^ PCE*,*^7^ SCORE,^8^ SCORE2,^9^ QRISK1,^40^ QRISK2,^31^ Prospective Cardiovascular Münster,^35^, ^36^ the WHO cardiovascular disease risk score,^10^ Globorisk,^31^ the INTERHEART modifiable risk score,^32^ Reynolds,^38^, ^39^ China-PAR,^37^ and PREVENT^13^, ^14^ show evidence of external validation. Most models show poor calibration among different ethnic cohorts, but acceptable discrimination. External validation of calibration is only relevant in a dataset that is truly representative of the contemporary target population. Risk scores that have demonstrated calibration in representative datasets or are indeed explicitly recalibrated to reflect risk in the target population include SCORE2,^8^ SCORE2-OP,^37^ the WHO cardiovascular disease risk,^10^ PREDICT,^11^ and PREVENT.^13^, ^14^

### Recalibration

Risk ratios (ie, model coefficients, such as hazard ratios or odds ratios) for established cardiovascular disease risk factors are generally similar across populations (ie, the association is similar across studies, countries, and time periods).^10^ Thus, good model discrimination is expected in new populations.^19^ However, models derived using cohort data can overpredict or underpredict risk in a target population either because the cohort(s) represent a past period with outdated event rates or the cohort(s) have distinct characteristics compared with the target population (eg, healthy volunteer bias or specific inclusion criteria). Electronic health records-based cohorts are often more representative of the target primary care population in which the risk score is applied, as compared with opt-in cohorts. Recalibration of cardiovascular disease prediction models following contemporary cardiovascular disease incidence rates and average risk factor values from the target population might be needed to minimise over-prediction and under-prediction of cardiovascular disease risk.^19^

Different recalibration methods exist, generally falling into two categories. For both, original risk ratios (coefficients) are retained, but the level of baseline absolute risk is reset to the target population. The first method, the replacement method,^55^ involves replacing the baseline survival (relating to the average population risk) and mean risk factor values with those of the target population. The second method, the regression method,^56^ involves splitting the target population into groups (eg, by predicted risk or age) and regressing the group-specific observed on predicted risks, yielding recalibration factors to bring predicted risks in line with those observed. In the regression method approach, group-specific predicted risks are obtained using observed risk factor values from the target population, entered in the uncalibrated model.

Traditionally, recalibration using either of these methods has involved using individual participant data from a contemporary representative cohort for the target population (eg, when the Framingham coronary heart disease risk model was recalibrated for Chinese populations).^57^ However, within the last decade, aggregate-level data have been used to recalibrate models.^9^, ^10^, ^31^ In this approach, annual age group-specific cardiovascular disease incidence estimates are extrapolated, providing 10-year expected risk estimates, and then used for recalibration, along with age group-specific risk factor averages that are used to obtain predicted risks. Using aggregate-level data for recalibration has some advantages over individual data. First, within many countries, aggregate-level data can be regularly and conveniently obtained from routinely collected data, avoiding reliance on costly and time-consuming accrual of individual-level prospective cohort data. Second, the use of aggregate-level data can facilitate capturing risk in the entire population, rather than relying on cohort data. Third, although this approach assumes that the single snapshot of 1 year can be extrapolated over a 10-year period, this might be a smaller assumption than the expectation that cohort data collected over a past period of 10 years (or often before) will be representative of the coming 10 years. Finally, routinely compiled data are often annually updated, allowing regular, rapid recalibration with changing trends in cardiovascular disease epidemiology.

The need for recalibration is context specific and depends on the current cardiovascular disease incidence rates in the target population and how similar these are to the rates in the dataset used for model derivation. Regular recalibration is likely to be needed where cardiovascular disease rates are rapidly changing. Some risk scores include an integrated framework to allow regular score updates (eg, Globorisk,^31^ WHO cardiovascular disease risk,^10^ and SCORE^8^ and SCORE2-OP^33^). The success of these approaches relies on the quality of the data used for recalibration. Data collection and quality vary enormously by country, with some countries having well established medical registries and personal identification numbers for all residents (linkable across different registers and yielding reliable cardiovascular disease incidence rates), whereas others have no medical record centralisation. For countries without internal registry data, relevant estimates are commonly available from international efforts to quantify disease incidence and risk factor values across multiple countries. For example, disease incidence estimates from the Global Burden of Disease study are available from the Institute for Health Metrics and Evaluation^2^, ^10^ and cardiovascular disease risk factors values from the Non-Communicable Disease Risk Factor Collaboration (NCD-RisC).

General practice electronic health records can provide advantages to ensure adequate calibration of risk scores corresponding to the observed incidence by using individual records combined with linkage to other national or hospital-based datasets. Although these records might not be fully representative of the general population, they can be considered representative of the users of primary care services. A synchronised approach in which models are embedded within general practice software for patient consultations could allow for a constantly evolving and improving risk prediction system. This approach can further support the monitoring of risk score usage, monitoring population observed and predicted risk distributions, assessing score calibration, and facilitating score recalibration. Some aspects of this approach have been used in the UK (QResearch database^40^) and New Zealand (PREDICT database^11^), although risk distribution and recalibration component monitoring have not explicitly been included in either country. Limitations of general practice electronic health records data in terms of accuracy and risk factor measurement completeness, as well as endpoint point ascertainment, should be carefully considered in line with any system development.

### Usability

Effective risk score use might improve clinician and patient risk perception and decision making (consequently improving patient outcomes), stimulate optimal medical resource usage, and reduce unnecessary costs and side-effects.^58^ Various tools have been developed to assist clinicians in cardiovascular disease risk estimation, interpretation, and communication^17^, ^59^ and the format of the tool can affect the uptake, accuracy of use, and resulting behaviour change or treatment decisions.^60^ Common formats include online calculators, mobile applications, simplified scoring sheets, colour-coded risk charts, and portable calculators. Colour-coded risk charts are considered easy to use, inexpensive, and can be used in locations without internet connectivity. However, such charts can only convey information for a few risk factors (often five to six are used) and their simplistic presentation often involves substantial rounding errors in risk estimates.^61^ Online calculators, such as HeartScore, give more accurate estimates, can be easily updated as necessary, and often provide graphical representation to improve communication.^8^, ^12^, ^41^ Integration of risk estimation systems within general practice databases has been shown to improve the usability of electronic calculators.^61^ For example, the integration of PREDICT^11^ in New Zealand resulted in a four-fold increase in cardiovascular disease risk estimates documented in medical notes.^61^

Approaches to further improve risk communication and impact are continuously developed. Suggestions include the use of heart age (also referred to as risk age), which provides an individual with the age of a person with identical estimated cardiovascular disease risk to them, but with optimal risk factors, aiming to motivate risk-reducing behaviour and treatment adherence in high-risk individuals.^62^

The use of cardiovascular disease risk scores varies globally, with various barriers to effective implementation including: reliance on laboratory measures,^21^, ^22^ low awareness of cardiovascular disease risk scoring, inadequate local or national government policy, general physicians fearing oversimplifying risk assessment by using prediction rules, concerns of overuse of medical therapy, poor patient compliance to recommended treatments, time restrictions, a belief that numerical risk estimates are not helpful for clinical decision making, and the presence of competing algorithms.^63^, ^64^ An integrated approach is needed to ensure optimal application and cardiovascular disease prevention, with clear locally relevant clinical guidelines, provision of effective estimation and communication tools, and adequate training for health professionals.

### Guidelines

Guidelines effectively bridge the complicated mathematical details of risk scores with understandable tools and treatment rules for clinical practice. Several global, regional, and national cardiovascular disease guidelines have evaluated and recommended cardiovascular disease risk scores for use in their respective target population. The guidelines can have marked differences in risk factors considered, recommended cardiovascular disease risk scores, tool formats, and thresholds recommended for categorising estimated cardiovascular disease risk to guide interventions.^4^, ^5^, ^16^, ^17^ These differences are considered acceptable as guidelines are based on localised and diverse populations, with recommendations for risk assessments based on their specific localised models being optimal when reliable information is available. Therefore, when choosing a risk score, it is important to explore which scores are guideline recommended for a relevant target population.

### Health gains and cost-effectiveness

Cardiovascular disease has a considerable impact on both health and social care systems, while also imposing a substantial economic burden on societies through enduring health costs and decreased economic productivity. In the EU, cardiovascular disease was estimated to cost €282 billion in 2021, with about €155 billion accounted for by health and long-term care, €48 billion by productivity losses, and €79 billion by informal care costs.^65^ Several population-based and individual-based cardiovascular disease prevention programmes, often requiring cardiovascular disease risk assessments, have been shown to be cost-effective across settings.^66^, ^67^, ^68^ Cost-effectiveness of cardiovascular disease prevention is dependent on many factors including baseline risk, population characteristics, cost of interventions, implementation of screening strategies, reimbursement procedures, and health-care budgets.^4^, ^66^, ^67^, ^68^ Therefore, cost-effectiveness is specific to a country or population, and, where possible, should be considered for localised settings (including local input parameters and cost-effectiveness thresholds).^4^

## Update on cardiovascular disease risk scores

The literature is saturated with research describing the development of new cardiovascular disease risk scores. Here, and in table 2 and table 3, we provide a summary of seven global or regional^8^, ^9^, ^10^, ^30^, ^31^, ^32^, ^33^ and nine national^6^, ^7^, ^11^, ^12^, ^13^, ^14^, ^34^, ^35^, ^36^, ^37^, ^38^, ^39^ key guideline-recommended risk scores used in different settings and summarise their characteristics according to the criteria listed in table 1.

### Description of guideline-recommended scores by geographical region

#### North and South America

One of the most widely used and reviewed risk scores is the Framingham, which is based on the pioneering US Framingham Heart Study from the 1960s.^6^ Other US scores were independently derived subsequently. For example, the Reynolds risk score^38^, ^39^ used the prospective follow-up of two randomised control trials for women^39^ and men^38^ separately, and the PCE^7^ (recommended by the American College of Cardiology–American Heart Association), combined eight prospective cohorts and has age-stratified and race-stratified models that estimate risk of atherosclerotic cardiovascular disease in White and Black adults.^7^ Although the PCE has been well validated internally and externally in terms of discrimination, and online tools exist for its implementation, most cohorts used in its derivation are from past decades (eg, baseline data from the 1980s or 1990s), with no recalibration to more recent cardiovascular disease event rates completed. Thus, the PCE might not reflect population-level changes in risk factor prevalence and preventive treatment in the contemporary era. Furthermore, the PCE might not be generalisable to individuals from racial and ethnic groups that are not included in the derivation data. The American Heart Association PREVENT model was developed in 2023 as a new sex-specific equation that includes estimated glomerular filtration rate as a predictor, includes heart failure as an outcome, and removes race from risk prediction estimates.^13^, ^14^ In comparison with the PCE, PREVENT was developed using a broader collection of data that is more representative of the current level of risk across the country and is therefore likely to present improved calibration as a result. Considering that race is a social construct and not a biological risk factor, race was removed in the clinical prediction algorithm, consistent with the overall consensus in medicine.^13^, ^14^

#### Europe

In Europe, the SCORE system was developed based on 12 pooled European cohorts to estimate the 10-year risk of fatal cardiovascular disease events for low-risk and high-risk populations, accommodating for some of the heterogeneity across Europe.^8^ Several countries recalibrated the SCORE model for their populations using individual risk factor data and local cardiovascular disease rates (eg, the Netherlands,^69^ Iceland,^70^ Greece,^71^ Switzerland,^72^ Türkiye,^73^ former Yugoslavia^74^).^8^ Recently SCORE2^9^ and SCORE2-OP^33^ have been developed and validated to estimate total cardiovascular disease risk (ie, fatal and non-fatal), with explicit recalibration to four European risk regions using aggregate-level data on expected incidences and risk factor distributions. The UK risk scores, QRISK1,^40^ QRISK2,^41^ and QRISK3,^12^ which are currently in use, differentiate themselves from the other scores by being developed on general practice attendee databases (ie, QResearch database). Similarly to the Framingham risk score, Scotland's ASSIGN score^34^ is based on an intermediate-sized cohort from the Scottish heart health extended cohort of the Scots study and, similarly to the QRISK models, includes a postcode linked index of social deprivation. Likewise, the Prospective Cardiovascular Münster risk score was developed from a prospective study in northwest Germany and estimates the risk of coronary heart disease and stroke separately.^35^, ^36^ Unlike SCORE,^8^ SCORE2,^9^ and SCORE2-OP,^33^ none of these country-focused risk scores have incorporated any recalibration to contemporary circumstances.

#### New Zealand and Australia

The New Zealand PREDICT risk score was developed using data from individuals who underwent cardiovascular disease risk assessments in health-care settings using the PREDICT software during primary care consultations (about a third of the country's population); the risk assessments were linked to national databases documenting drug dispensing and hospital admissions.^11^ In Australia, a modified version of the PREDICT equation, which has been recalibrated for the Australian population,^75^ is currently being recommended by the 2023 Australian guideline for assessing and managing cardiovascular disease risk.^76^

#### Asia and Africa

Although cardiovascular disease risk scores derived from predominantly White populations (mostly from Europe and the USA) have been well established, risk scores for other ethnic populations are either absent, under-reported, or unvalidated.^22^ In the most populous continent, Asia, only a few cardiovascular disease prediction models have been established based on large cohorts in Korea,^77^ Singapore,^78^ Thailand,^79^ India,^80^ Israel,^81^ China,^37^, ^57^, ^82^ Japan,^83^ and Iran.^22^, ^84^ For example, China-PAR was developed based on two prospective Chinese cohorts (ie, interASIA and China MUCA) and included risk factors, such as urbanisation and geographical region to improve discrimination.^37^ To our knowledge, no specific risk score for African populations has been developed yet.^22^

#### Global

To adapt cardiovascular disease risk predictions to wider geographical regions and LMICs, WHO supported the development of the WHO cardiovascular disease risk score, which is recalibrated according to contemporary risk factor levels and disease incidences across 21 global subregions.^10^ Compared with the previous WHO–International Society of Hypertension score, the WHO cardiovascular disease risk score allows for flexible recalibration as new epidemiological data emerge and enables more accurate risk estimation in different settings.^10^, ^30^ Another global score, Globorisk,^31^ provides 10-year cardiovascular disease risk assessments for 182 countries. Lastly, the INTERHEART modifiable risk score is based on the INTERHEART case-control study and uses multiple modifiable risk factors applicable to an international population, but does not estimate absolute risk.^32^

### Population and patient-specific risk scores

Although this Review focuses predominantly on cardiovascular disease risk scores used in general populations for primary prevention across different geographical settings, several population-specific or patient-specific risk scores exist (although these have predominantly been developed in Europe). For example, SCORE-OP estimates cardiovascular disease risk in individuals aged 65–79 years;^33^, ^85^ SCORE2-Diabetes, ADVANCE,^86^ DIAL1/2,^87^, ^88^ and a diabetes-specific PREDICT^11^ are designed to predict cardiovascular disease risk in people with type 2 diabetes; SMART estimates risk for recurrent atherosclerotic cardiovascular disease in patients with established atherosclerotic cardiovascular disease;^89^ and MAGGIC estimates risk of heart failure among patients with previous heart failure that have preserved ejection fraction.^90^ Each score incorporates risk factor associations relevant to the specific subgroup of the population, as well as additional risk predictors that provide context-specific information. The criteria presented in table 1 and earlier in this Review are equally relevant for these population subgroup-specific risk scores in terms of model development and the potential need for recalibration. Recalibration can, however, be challenging due to difficulties in obtaining cardiovascular disease incidence rates for the specific population subgroups from routine registry sources.

## Conclusion

Key steps and criteria for determining if a given cardiovascular disease risk score is appropriate for use in a particular population are outlined in the figure, with an example in the panel. The steps focus primarily on the suitability of the risk score given characteristics of the target population for screening (eg, geographical location, sex, age) and whether the risk score is appropriately calibrated for the target population, as well as practical considerations (eg, the feasibility of laboratory measurements). An integral assumption of this process is that the risk score being considered will also meet the derivation and validation criteria outlined in table 1.

Panel **Example assessment of candidate cardiovascular disease risk scores for use in Bangladesh**

A 10-year overall (ie, fatal and non-fatal events) cardiovascular disease risk prediction model is required for use in Bangladesh. Following the steps in the figure:

**Figure:**
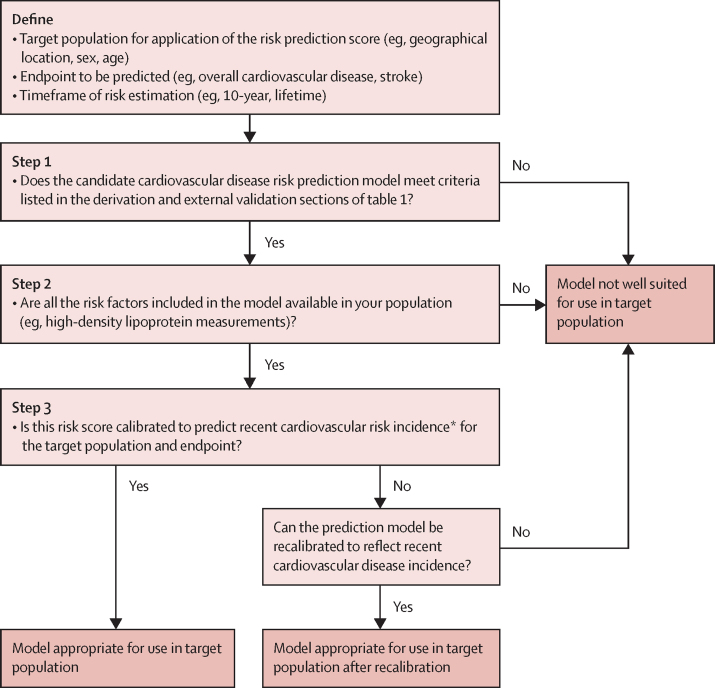
Steps to determine if a candidate cardiovascular disease risk prediction model is appropriate for a given population of interest Where more than one candidate CVD risk model is deemed appropriate for use in a population, preference should be given to any score that is recommended by national or international guidelines and used in accordance with guideline recommendations. *Reflecting current cardiovascular disease incidence rates and epidemiological trends in the population; target populations with rapidly changing cardiovascular disease rates will need more frequent updates (for example every 5 to 10 years).

• The adult primary prevention population of men and women aged 40–80 years in Bangladesh is the target population
• Step 1: candidate cardiovascular disease risk scores for this geographical region that meet the table 1 derivation and validation criteria are the WHO cardiovascular disease risk score and Globorisk

**Table 1:** Checklist for cardiovascular disease risk prediction models in clinical practice

	**Considerations**
**Derivation**	
Population	Does the dataset used for model derivation contain information on a sufficient number of individuals and events (ie, is the sample size large enough)? Are the characteristics of individuals in the derivation sample sufficiently aligned with those of the target population for risk assessment to allow transferability of estimated risk ratios (eg, age range, previous disease status)?
Risk factors	Are the relevant risk factors included? Inclusion of major lifestyle risk factors (eg, smoking, blood pressure, and BMI or cholesterol) should be a minimum. Are the included risk factors accurately measured in sufficient numbers in the derivation sample? For example, use of continuous blood pressure is more accurate than classification of hypertension based on measured values or self-reported history. Can all risk factors be feasibly measured and are alternative formats available for resource-constrained settings? For example, risk scores using risk factors that do not require laboratory measurements might be more feasible in some settings.
Endpoints	Does the risk score predict the relevant endpoint (eg, overall cardiovascular disease, stroke, coronary heart disease)? Is endpoint collection in the derivation sample systematic and well validated?
Follow-up	Is the follow-up time in the derivation sample sufficient to allow risk estimation over the timeframe of interest (usually 10 years)?
Statistical model	Are appropriate statistical models used for the type of risk estimation? Are relevant assumptions tested (eg, proportional hazards assumption)?
Internal validation	Discrimination—is the model able to predict order of cardiovascular disease events among individuals? Calibration—is there good agreement between predicted and observed incidence (absolute risk). Has the model been checked for overfitting and optimism in performance? Overfitting is particularly relevant for smaller sample sizes and can be checked by cross-validation or shrinkage. Reclassification—is there appropriate movement of individuals through relevant risk categories when compared with alternative risk models?
**External validation**	
Transferability	Is the model proven to be transferable to the target population in which the score is being used? For example, does the model have a good predictive performance (eg, discrimination and calibration) when applied to new target individuals from a data source that was not used in the model development?
Recalibrated	Has the model been appropriately recalibrated for use in the target population?
Recalibration data	Are the data used for recalibration appropriate? For example, do the recalibration data share the same characteristics as the target population?
Recalibration methods	Is there a methodological framework proposed or provided for future recalibration in response to changing trends with time as well as divergent cardiovascular disease rates across regions and populations? For example, is a guide or statistical code provided for recalibration? What is the ease of recalibration? Are a lot of extra resources required? Are additional data needed for recalibration and are they available?
**Usability**	
Format	Is the format appropriate to be used in the population to which the model is applied (eg, online risk calculator, colour-coded charts)?
**Implementation**	
Guidelines	Has the model been recommended for use by relevant guidelines? This can be either national, regional, or global.
Health gains*	Has the risk score been evaluated for health gains when used to assess cardiovascular disease risk and guide interventions (eg, statins) for high-risk individuals? Has use of the risk score resulted in significant health gains when used?
Cost-effectiveness*	Has use of the risk prediction model shown to be cost-effective?

If of interest, the TRIPOD guidelines can be used for a more detailed checklist for statistical risk prediction modelling.

* Cost-effectiveness and health gains of (different) cardiovascular disease risk models in clinical practice are highly dependent on various factors, such as the target population, baseline cardiovascular disease risk, accompanied clinical guidance (eg, statin allocation), the cost of the drugs or other interventions, reimbursement procedures, and implementation of preventive strategies. Assessments of the health gains and cost-effectiveness might also not be available in many countries and should be considered where available.
• Step 2: there are scarce resources to provide laboratory measurements in some target areas; both laboratory and non-laboratory versions of both the WHO cardiovascular disease risk score and Globorisk are available; the non-laboratory versions only require measurement of age, sex, systolic blood pressure, smoking, and BMI, all of which are available in the target population
• Step 3: both risk scores have been calibrated to predict total (ie, fatal and non-fatal events) cardiovascular disease risk using incidence estimates from within the past 5–10 years in the target population, Globorisk specifically for Bangladesh and the WHO risk model for south Asia
• Both risk scores are appropriate for use; preference might be given to the WHO risk score since there are specific recommendations regarding its use in WHO guidelines

In conclusion, there are various national, regional, and global risk scores available for cardiovascular disease risk estimation, but they differ in derivation methodology, included risk predictors, endpoints predicted, and derivation and target populations. To identify an appropriate cardiovascular disease risk score with good predictive ability, including discrimination and calibration, health professionals and policy makers need to compare and select risk scores based on the needs of cardiovascular disease prevention as well as the practicality of use in their local population. In the vast majority of cases, regular recalibration of the risk score is needed to ensure that the estimated risks reflect those in the target population, and this can be completed using aggregate-level data. The use of a cardiovascular disease risk score that includes recalibration as an integral process is advantageous.

### Search strategy and selection criteria

To inform this Review, we searched PubMed, Embase, Google, and Google Scholar to identify existing risk prediction models for cardiovascular disease in the context of primary prevention recommended by global, regional, and subregional guidelines published in English, from database inceptions to January, 2024, using relevant terms, such as “cardiovascular disease”, “risk score”, “risk equation”, “risk algorithm”, and “risk prediction”. The intention was not to provide an exhaustive review of all published cardiovascular disease risk scores, but to give a contemporary description of several selected scores, which are recommended by prominent clinical guidelines for primary cardiovascular disease prevention, while also exemplifying key issues relating to risk score development, application and valuation, and selection for use in the given target population.

### Contributors

## Declaration of interests

SK reports grants from British Heart Foundation, grants from National Institute for Health and Care Research, and grants from Health Data Research UK, during the conduct of the Review. All other authors declare no competing interests.
